# SEXUAL ORIENTATION AND THE ONSET AGE OF CHRONIC DISEASES AND DISABILITIES

**DOI:** 10.1093/geroni/igae098.0522

**Published:** 2024-12-31

**Authors:** Jen-Hao Chen

**Affiliations:** National Chengchi University, Taipei, Taipei, Taiwan (Republic of China)

## Abstract

One key to increasing healthy lifespan and reducing health disparities is delaying the onset of chronic conditions and associated disabilities, especially among marginalized groups. Despite this understanding, relatively few research studies investigate the onset age of chronic diseases and disabilities by sexual minority. This study analyzed pooled data from the 2013-2019 U.S. National Health Interview Survey (NHIS) to investigate the relationships between sexual orientation (i.e., heterosexual, homosexual, bisexual, and other sexual minorities) and the onset of chronic diseases and disability. The study first calculated the onset age for a wide range of chronic conditions and disabilities (i.e., vision and hearing problems, hypertension, diabetes, cancer, intellectual disabilities, etc.), then used weighted regressions to link sexual orientations to the age of chronic disease and disability onset, controlling for a wide range of sociodemographic characteristics. Results suggest that there are considerable disparities in the onset age of chronic diseases and disabilities between heterosexual individuals and sexual minorities. Bisexual and homosexual individuals were significantly more likely to have chronic diseases and disabilities earlier in life than their heterosexual counterparts, with bisexual individuals being the most disadvantaged. These disparities were not accounted for by the higher education and income levels observed in some sexual minority groups. However, there was no difference in terms of disease and disability onset between heterosexual individuals and other sexual minorities. Some cohort differences were found. Given the historical discrimination against sexual minorities and challenges related to HIV, these findings underscore the need for policy interventions to improve sexual minorities’ healthspan.

